# The Importance of Reverberation for the Design of Neonatal Incubators

**DOI:** 10.3389/fped.2021.584736

**Published:** 2021-04-07

**Authors:** Virginia Puyana-Romero, Daniel Núñez-Solano, Francisco Fernández-Zacarías, Edgar Jara-Muñoz, Ricardo Hernández-Molina

**Affiliations:** ^1^Grupo de Investigación Entornos Acústicos, Facultad de Ingeniería y Ciencias Aplicadas, Universidad de Las Américas, Quito, Ecuador; ^2^Laboratorio de Ingeniería Acústica, Universidad de Cádiz, Cádiz, Spain; ^3^Unidad de Neonatología, Hospital Metropolitano, Quito, Ecuador

**Keywords:** reverberation time, neonates' well-being, neonatal incubator, acoustic environment, low frequency noise

## Abstract

Low frequency noises are predominant in neonatal intensive care units (NICUs). Some studies affirm that neonates can perceive noises from 113 Hz, and can therefore be affected by sound sources with high spectral content at low frequencies (e.g., incubator engine, air fan). Other studies suggest that reverberation amplifies noise within incubators. In this paper, the reverberation time (*T, T*_30_) within an incubator with standard dimensions was measured in one-third octave bands. To get reliable results, the *T* was measured in 15 positions at the neonate's ear height, in a room with low *T* values (to reduce the influence of the room in the results), using an impulsive sound method. Results show a heterogeneous *T* distribution at the neonate's ear height, with maximum average *T* differences between positions of 1.07 s. The highest average *T* of all microphone positions is 2.27 s at 125 Hz, an extremely high mean value for such a small space. As the frequency of electrical devices in America is 60 Hz, some harmonics lay within the one-third octave band of 125 Hz, and therefore may create a very reverberant and inappropriate acoustic environment within the audible spectrum of neonates. As the acoustic environment of the incubator and the room are coupled, it is expected that the results are higher in the NICUs than in the room where the measurements were conducted, as NICUs are more reverberant. Therefore, it is recommended that the *T* will be limited in the international standards, and that incubator designers take it into account.

## Introduction

The neonatal incubator provides protection from the exterior environment, optimal temperature and air renovation conditions for the development of the newborn, and it is intended for pre-term, late pre-term, term, post-term, or sick children. However, the electrical devices that maintain these optimal conditions generate high noise levels. The noise level inside the incubator caused only by the engine running ranges from 52.3 to 56.7 dB (1). Besides, there are also other equally important noise sources that may alter the neonate's rest within the neonatal intensive care unit (NICU), such as the air ventilation system or the healthcare team (2, 3). Although these noise levels have been reduced in recent years, noise within incubators are still far from the 45 dB recommended by the American Academy of Pediatrics for neonatal care areas (4–7).

The noise spectrum inside the uterus is different from the one of the NICU (8, 9), which may have adverse effects on the pre-term infant auditory system; as the womb protects the child from high frequency noises, some studies suggest that the auditory system of pre-mature infants is not mature enough to process the noisy environment of the NICU. This may increase the risk for auditory, language, and attention disorders in the future (10, 11).

The spectral content of NICUs shows a predominance of low frequencies (3, 12, 13), except for some mid-high frequency events caused by the equipment alarms and human voices (2). Some studies reveal that newborns can perceive sound stimuli from 113 Hz onwards (14), (15). However, the information about the specific effects of the low frequencies exposure on neonates has been hardly studied. In that regard, a recent study suggests that if low-frequency exposure has adverse effects on animals that have a higher threshold at low frequencies than humans, it could also have negative effects on humans (16). In the case of mice, exposure to low-frequency noise of 100 Hz at 70 dB for 4 weeks causes them permanent imbalance (16), even though they cannot hear sounds below 375 Hz (17).

Noise may also interfere with the development of the nervous system and subsequent behavior of the neonate (18, 19). Among others, its adverse effects are related to the evolution of the auditory perceptual skills [e.g., sound localization problems, speech discrimination with high background noise, differentiation of sound frequencies (20), audition loss (21, 22), and interference for pattern recognition (20)]; alterations of the vital signs [heart rate (19, 23), oxygen saturation (24), respiratory rate (19, 25), blood pressure (26)] and neonate's rest; and negative psychological effects (19, 27).

Therefore, the noisy environment that comes both from outside and inside the incubator is potentially harmful to the future development of the neonate. The noises emitted (e.g., by the incubator engine, monitoring alarms, or the air fan) may be modified because of an acoustic phenomenon that occurs on reflective surfaces. This phenomenon is called reverberation and may cause persistence, distortion, and amplification of sounds. A reverberant room, like the incubator, can increase the sound power of the noise emitted, as a function of reverberation time (*T*), at quite significant levels. The higher the *T*, the worse its effects. Some research, which suggests that the reverberant walls of the dome amplify noise within the incubator, have measured higher noise levels with closed doors than with open doors (28, 29). In this sense, the only research article found that studied the *T* inside the incubator showed that the *T* of the portholes opening and closing operations were between 2.0 and 3.7 s (30); however, some important procedural aspects were missing in the description of the measurements (e.g., number of measurements, or microphone location during the measurements).

ISO 3382-2 (31) is normally used to conduct *T* measurements in ordinary rooms. Nevertheless, some difficulties may be encountered by applying the cited standard to small spaces, especially to fulfill the recommended distances between microphones and source positions (32).

Another phenomenon that can generate sound amplification may occur when the emitted sounds contain resonant frequencies of the room. The axial vibration modes of rooms are excited by waves that propagate between two parallel surfaces: when the distances between these two surfaces are equal to half a wavelength (λ/2) or odd multiples of λ/2, a modal resonance appears. Two pairs of parallel surfaces are responsible for the tangential vibration modes, while three pairs of parallel surfaces will form oblique modes. Modal resonances cause a heterogeneous energy distribution in the room, which may also lead to a heterogeneous *T* distribution (33). The effect of resonant frequencies is especially notable at low frequencies-being the effect of the lowest resonant frequency (fundamental frequency) the most pronounced. Therefore, the identification of the low, frequency range in small enclosures, which can be done by calculating the Schroeder frequency (34), is very important.

### Objectives

Since the noise present inside the incubator, as a consequence of an external or internal source, can be amplified by the reverberation phenomenon, the objective of this paper is to analyze the behavior of reverberation inside the incubator, without the influence of the NICU. Consequently, the reverberation time was measured within an incubator, in a room with controlled acoustic conditions.

The small dimensions of the incubators could be a disadvantage at low frequencies, as the effect of the resonant frequencies may cause substantial variations of the *T*s at different points of the room.

## Methods

**Figure 1:**
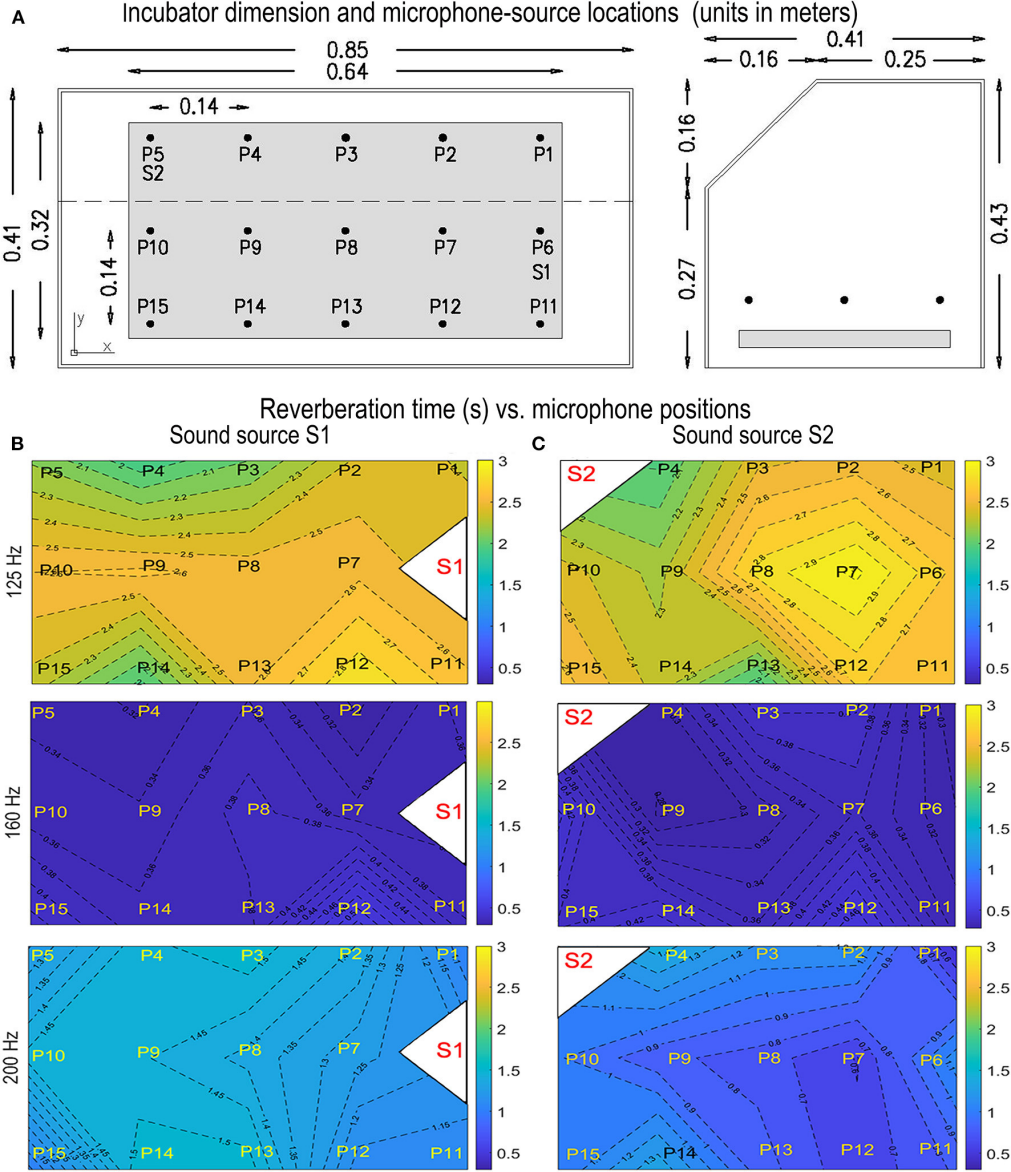
**(A)** Incubator dimensions, microphone locations P1 until P15, and sound source locations S1 and S2. The thickness of the incubator walls is 6 mm. Note that when the sound source is at S1, the microphone location P6 was not used. The same happened with S2 and P5. **(B)** Mean *T*_30_ at 125, 160, and 200 Hz -sound source at position S1 and S2 **(C)**, for the 15 microphone positions. The blue color shows the positions with the lowest *T*, and the yellow ones, the position with the highest *T*.

*T* measurements were carried out using 15 microphone positions inside a YP_90A incubator model Ningo David Brand. Microphones were placed on a horizontal plane approximately at the newborn's ear height. Measurements were conducted with the mattress inside the incubator. The *T* was estimated by the *T*_30_ (a good indicator if “Max level”–“Background level” ≥45 dB), which measures the time that the sound pressure level (SPL) decays 30 dB after the first 5 dB of decay. *T* measurements were carried out by locating a sound source inside the incubator at two different positions, S1 and S2. Fourteen measurements were conducted for each sound source position, one less than the microphone positions because one of the positions was occupied by the sound source. The incubator has three pairs of parallel surfaces and a chamfer at the top. The dimensions of the incubator and the microphone locations are shown in Figure 1A.

The room in which the acoustic measurements were conducted is covered with sound-absorbent materials (with mean *T*_100−5, 000*Hz*_ below 0.15 s; see **Figure 3** for detailed information on the *T* of the lowest frequencies studied), to reduce the influence of the room in the results. The room dimensions are 1.79 × 3.87 × 2.45 m.

An impulsive sound was generated by bursting a balloon to measure *T*. Although it is a sound source that presents certain drawbacks, such as directional sound radiation at some frequencies (35–37), it was chosen because its size can be adapted to the size of the incubator, and because it matches the conditions of the ISO 3382-2 for omnidirectional sources at frequencies above 500 Hz (35). To get uniform impulsive sounds, balloons of similar diameter, brand, color, and air inflation volume were burst (35, 37, 38). No criterion was found in the literature for the number of sound source positions used for small enclosures: It was decided to use two sound source positions, meeting the recommendations of the ISO 3382-2 for the engineering level of measurement accuracy. No recommendations are given by the named ISO regarding the location of the sound sources. One was located in a corner, to excite standing waves easily (39) and the other was located next to where the baby's head might be (Figure 1A).

A CESVA class 1 calibration pistonphone was used to validate the measurement chain, with checks at the beginning and the end of the measurements. An external AVID sound card connected to a PC and a GRAS microphone were used for noise measurements. For each measurement, an impulse response audio file was stored, using a sampling frequency of 44.1 kHz.

A total of 1,120 measurements were made, with 40 decays at each point. The ITA toolbox (40) and Matlab were used for post-processing data to get the *T*_30_. The analysis was carried out in one-third octave bands, from 100 until 5,000 Hz.

A compromise was looked for when measuring, to avoid the influence of the measurement room. The two portholes on the sidewalls were kept partially closed, but the holes used for introducing breathing tubes or heart monitor electrodes cables were not, since they are not that prominent in comparison with the surfaces of the incubator dome. Instead, these small holes were used to introduce the microphone cable and the balloon bursting tool.

To verify if there is a heterogeneous *T* distribution at the different frequencies analyzed, the Kruskal Wallis test was conducted for all the microphone-source combinations. The test was applied independently to each one-third octave band. Kruskal Wallis-test (41) is a rank-based non-parametric-test that determines if there are statistically significant differences between at least two categories of the independent variable (in this study, the categories are the *T* values at each microphone position). However, it does not indicate which of the categories of the independent variable are statistically significantly different from each other. To do that, the Mann Whitney-test (42) was conducted on the data of each one-third octave band for all the possible paired combinations of microphone positions. Positions P5 and P6 were discarded from the tests because the sound source occupied these positions.

A brief study on the vibration modes of the incubator was reported, in order to understand what happens at the frequencies with the highest *T*.

## Results

Table 1 shows the mean *T* for each sound source position. Results reveal that the two highest values occur at 125 and 200 Hz, with mean values of 2.27 and 1.08 s, respectively. From 200 to 400 Hz, the *T* decreases as the frequency increases. However, from 500 Hz, there is a slight *T* increment up to 1,250 Hz and a subsequent decrease onwards.

**Table 1:** Statistical data—standard deviation, mean, median, Kruskal-Wallis-test and Mann Whitney-test-conducted on the reverberation time.

**Frequency (Hz)**	**Sd**	**Mean (s)**	**Median (s)**	**Kruskal Wallis** ***P*-value**	**Mann Whitney** **number of pair of positions (among 79 possibilities) with significant differences. *P*-value < 0.05**
100	0.57	0.95	0.74	0.000	23
125	0.89	2.27	2.31	0.000	26
160	0.25	0.37	0.30	0.000	25
200	0.52	1.08	1.16	0.000	47
250	0.15	0.23	0.18	0.000	28
315	0.10	0.20	0.18	0.000	43
400	0.06	0.13	0.12	0.000	23
500	0.03	0.15	0.15	0.000	37
630	0.02	0.17	0.16	0.000	51
800	0.02	0.17	0.17	0.000	45
1,000	0.02	0.18	0.18	0.000	29
1,250	0.02	0.19	0.19	0.017	10
1,600	0.02	0.19	0.19	0.000	19
2,000	0.02	0.19	0.19	0.001	21
2,500	0.02	0.19	0.18	0.000	28
3,150	0.02	0.18	0.18	0.000	36
4,000	0.02	0.17	0.17	0.000	36
5,000	0.02	0.17	0.17	0.000	41

Kruskal Wallis results show that there are statistically significant differences between two or more microphone positions along the bands frequencies analyzed, with a level of significance below 0.001% at all frequencies but 1,250 and 1,600 Hz, in which the level of significance was below 0.050%. Mann Whitney-test reveals that in all the frequencies analyzed, there are at least 10 pairs of positions with different *T*. The frequencies with more positions statistically different are 200 and 630 Hz, with 47 and 51 pairs of positions with different *T*, respectively, among 79 possibilities.

The fundamental frequency of the air cavity is at 202 Hz. It happens for the resonant mode m1, considering the X axis at the largest dimension of the incubator (see Figure 1A). Other resonant frequencies, sorted in increasing order, are *f*_m2_ = 404 Hz, *f*_m3_ = 419 Hz (generates the lowest resonant mode at the Y axis), *f*_m4_ = 429 Hz (lowest resonant mode at the Z axis) or *f*_m5_ = 606 Hz. The fundamental frequency is suggesting that there is no reason for having high mean *T*s below 202 Hz; however, there is a noticeable increase in the *T* at 125 Hz. That can be explained by the influence that the structural resonances may have in small places with thin reflective walls like the incubator, and also by the acoustic impedance of the incubator dome material at low frequencies.

The Schroeder frequency (34) is 3,143 Hz, considering an average *T*_30_ of 0.37 s and a volume of 0.15 m^3^. According to the criterion used in room acoustics, below the Schroeder frequency the resonant frequencies may cause a heterogeneous spatial distribution of the acoustic pressure. It is worth highlighting that small variations of the *T* for rooms with small volumes involve high changes in the Schroeder frequency calculated. The Schroeder frequency obtained should not be taken in an alarmist way: considering the wavelength of the resonant frequencies close to that value, there would be planes of maximum and minimum acoustic pressure at distances of ~0.03 m (λ/4); given the small distances, we consider that pressure changes would hardly be appreciated. It is difficult to identify which distances between maximum and minimum pressure values (and therefore which resonant frequencies) can be appreciated by a neonate. However, the acoustic environment perceived inside the incubator will surely be very different from that perceived outside because of the effect of amplification that the acoustic resonances may have.

Figures 1B,C show the average *T* measured for the 15 microphone positions at three third-octave bands 125, 160, and 200 Hz. These third-octave bands were chosen to compare low frequencies with quite different *T*-values. Figures 1B,C show the average *T* for the sound source positions S1 and S2, respectively. When the sound source is at the S1 position, no measurement was made at the P6 microphone position. The same happens with positions S2 and P5 (Figures 1B,C). When the sound source is at position S1, the largest *T* differences between positions vary from 0.20 to 1.07 s at 160 and 125 Hz, respectively. There is a heterogeneous *T* distribution at all the frequencies analyzed. No pattern can be appreciated for the *T* distribution at 160 Hz or at 125 Hz for any of the two sound source locations used. Since the 200 Hz frequency band contains the lowest resonant frequency of the air cavity, it was expected that the effect of resonance was reflected in a *T* distribution with a recognizable shape. When the sound source is in S1, higher *T*s can be observed at P3, P4, P9, P13, and P14, although further analysis is needed to get more consistent results. For S2, this effect cannot be appreciated, probably because the proximity of the sound source to the corner may have excited better the structural resonances, the effect of which is reflected in the spatial distribution of *T*.

**Figure 2:**
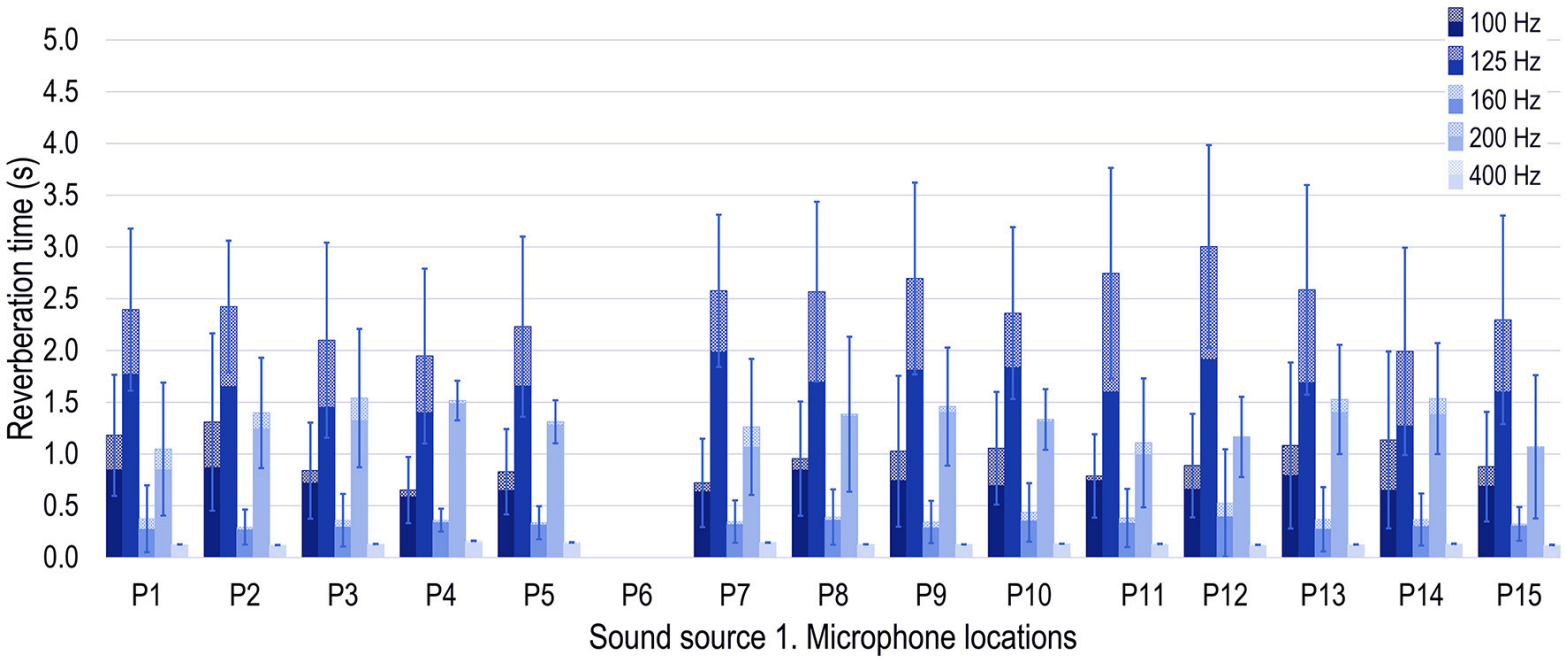
Median (solid hatch pattern) and mean (dotted hatch pattern) values at the 15 microphone positions at 100, 125, 160, 200, and 400 Hz. Error bars show the standard deviation of the sample. Note that when the sound source is at S1, the microphone location P6 was not used.

To evaluate the influence of the fundamental frequency, and also to look for geometric patterns in the *T* distribution, the median values have been represented at the 200 Hz band (Figure 2 light orange line). Median values were expected to offer more robust results, and to reduce the source proximity effect. Note that, as it happened in Figure 1, when the sound source is at S1 the microphone location P6 was not used. If three groups of microphone positions parallel to the X axis are considered, P1 to P5, P6 to P10, and P11 to P15, it can be observed that the TRs at the central positions P8 and P13 are higher than the ones at the lateral positions. To find bases that explain the observed *T* distribution at 200 Hz, the research conducted by Horvat et al. (33) was examined. They analyze the *T* in octave bands in a room with a volume of 152.50 m^3^ (in contrast, the incubator volume is ~0.15 m^3^). They affirm that there is a relationship between SPL and *T* values caused by the resonant frequencies with predominant effects; in the figures shown in the cited paper, high SPLs correspond to the proximities of low *T*, and vice versa. Although they measure in rooms where structural resonant frequencies can be neglected, and probably in the incubator, they cannot, some conclusions can be extrapolated for the 200 Hz band; for the first resonant mode, lower SPL are expected at positions P3, P8, and P13, were the nodal plane is. According to the cited paper, these positions should have the highest *T* values, as it happens in the incubator for positions P8 and P13. Position P3 is the second highest of the P1–P5 which can be appreciated in Figure 2. Higher *T* values can be observed also in positions P7, and P9; therefore, at 200 Hz, the positions with highest *T*-values are near were the neonate's ear can be.

The second resonant frequency of the air cavity, 404 Hz, belongs to the 400 Hz one-third octave band. As this band frequency has mean *T*-values below 0.25 s the *T* spatial changes can be hardly appreciated (Figure 2). No pattern on the spatial distribution of the *T* can be found from 100 until 160 Hz. The high standard deviation (Sd) of the measurements at low frequencies, the proximity effect of the sound source, the asymmetric shape of the incubator regarding the XZ and the XY planes, and the existence of different structural resonances acting at the same time in the frequency band considered can make it difficult to find any pattern in the *T* distribution. Although no pattern was found, these *T* distribution changes make the acoustic environment unnatural.

**Figure 3:**
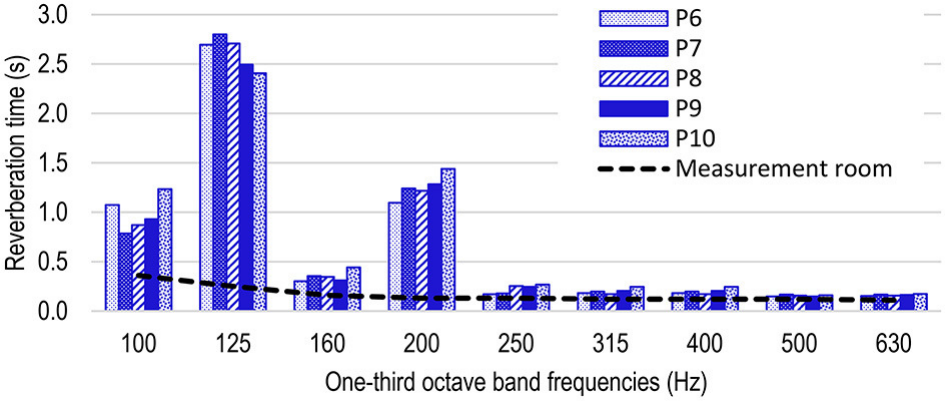
Mean *T* at positions P6, P7, P8, P9, and P10, considering both sound source positions (S1 and S2). Mean *T* of the measurement room according to ISO 3382-2 (black line).

Figure 3 compares the *T* values in the possible locations of the newborn's ear (P6, P7, P8, P9, and P10), which allows evaluating whether this lack of uniformity in the *T* distribution occurs in these positions. For the sake of clarity, Figure 2 shows only the *T* from 100 to 630 Hz. At those frequencies, the *T*s of the measurement room are also shown. Although not all the possible locations of neonate's head were measured, these five points can be considered as references to evaluate the *T* distribution. The *T* differences are considerable, especially bearing in mind that they are average values, and happen at a distance of just 14.00 cm. The *T* differences between the five microphone positions are higher at low frequencies. The higher differences are at 100, 125, and 200 Hz, with values of 0.29, 0.39, and 0.26 s, respectively. On the contrary, the differences can hardly be appreciated above 400 Hz. On the other hand, at 125 Hz, the *T* levels at all the positions are above 2.40 s. Figure 3 allows the comparison of the *T*s inside the incubator with the *T*s of the room. Although the influence of the room cannot be ruled out, it can be concluded that the high *T*s at low frequencies are mainly caused by the incubator.

## Discussion

In this paper, the *T* distribution was evaluated inside a neonatal incubator. The incubator was located in a room with a low *T* to reduce the possible influences of the measurement room. Acoustic measurements were performed in a plane at the height of the newborn's ear. Results reveal that there are statistically significant differences between the *T* measured at the different positions. These differences are remarkable especially at low frequencies, even if just the possible locations of the neonate's head are considered.

The *T* is proportional to the space volume, and the incubator volume is below 0.15 m^3^. Therefore, the *T* measured may be considered normal for ordinary rooms, but for such a small space, it is incredibly high, especially at low frequencies. The *T*s of the measurement room reveal that the high *T*s inside the incubator at low frequencies are not due to the room but to the incubator. Therefore, in this experiment, the influence of the resonant frequencies of the room can be considered negligible. However, the resonant frequencies of rooms with acoustic reflective surfaces -like NICUs- may influence the *T* of the incubator, especially considering that the acoustic insulation of the incubator is very poor (43, 44).

The lack of uniformity of the *T*s distribution at frequencies within the typical acoustic spectrum of a NICU and their high duration reveal an unnatural acoustic environment. At 125 Hz, the *T* levels of the possible neonate's head positions are above 2.40 s. As the electronic devices in America operate at 120 V, with a frequency of 60 Hz, their harmonics may generate a heterogeneous energy distribution at 125 Hz one-third octave band, and therefore, create a very reverberant and inappropriate acoustic environment for the neonate.

The mean *T* of the incubator at 250 Hz is 1.08 s. The fundamental frequency of newborns cry is in the range of 200–500 Hz (45); the noise generated by medical staff shifts (>50 dB) is between 15 and 4 kHz (13); and the one generated by continuous positive airway pressure systems is between 25 and 6.3 kHz (46). Therefore, there are activities in a NICU that generate sounds within the frequency range in which resonance effects cause high *T* levels.

In a study conducted by this research team on three neonatal incubators in a controlled acoustic environment, noise levels were measured inside the incubators with the engine on, obtaining maximum SPLs of 46.2 dB at 100 HZ, 47.0 dB at 125 Hz, 43.1 dB at 160 Hz and 45.9 dB at 200 Hz. (47). When the incubators were in the NICU, levels were measured inside (int) and outside (ext) the incubator, obtaining SPLs of 60.0 dB-int and 48.0 dB-ext at 100 Hz, 53.6 dB-int and 46.8 dB-ext at 125 Hz, 57.5 dB-int and 49.5 dB-ext at 160 Hz, and 56.0 dB-int and 52.7 dB-ext at 200 Hz. These results show that, at these frequencies, the noise inside the incubator is higher than in the NICU room (48). It can be affirmed that the noise inside the incubators when they are in the NICUs -at the problematic frequencies in our study- is very high, largely due to the effect of reverberation. If incubators were designed to reduce this effect, indoor noise levels would be lower. Although the solution to the problem is not simple, the placement of acoustic absorbent materials that meet hygienic-sanitary conditions for this type of space and allow to keep the eye-contact with the neonate, or the use of less reflective transparent materials and non-parallel walls could reduce the problem.

The highest Sd-values are at low frequencies. This can happen because, for all balloon types, the SPL deviations below the 500 Hz octave band are on the order of 6.0–9.0 dB (35). Therefore, below 400 Hz one-third octave band, some measurements may lead to an appropriate signal-to-noise ratio when measuring the *T*_30_ and some others not, and that may one reason why the Sd at low frequencies is high.

Although there are different incubator models on the market, a high number of them have similar proportions and are constructed with materials with similar reverberant features. If we consider double wall incubators, the inner space dimensions in which the neonate is placed are similar to the ones studied in this research; furthermore, the effect of the double wall may also originate inner resonances between both walls, which could generate an inappropriate environment from an acoustic point of view. Hence, we consider that the results can be transferred to other incubator models, at least in the existence of heterogeneous energy and *T* distribution inside the incubator, although there may be slight differences in the *T* values for the frequency bands considered.

The reverberation phenomenon within incubators is not specified in the standards ANSI/AAMI/IEC in which the compliance requirements for incubators are defined. As a general reflection on the *T*s obtained, it is worth mentioning the requirements/recommendations established by different European countries on reverberation in hospital wards. Countries such as Sweden, Denmark, Finland, France, Norway and Poland set maximum *T* values for this type of room ranging between 0.50 and 0.80 s (49). Bridging the gaps with the standards on room acoustics, the mean *T* at certain frequencies obtained in this research is very high (*T*_125Hz_ = 2.27 s), and we believe that the standards on neonatal incubators should include limitations regarding the *T*.

## Conclusions

The interiors of incubators are quite reverberant spaces. The results of this study show very high *T* at low frequencies considering the small volume of a neonatal incubator. The area with the highest reverberation is around the mattress centerline, right where the newborn is placed. Therefore, the incubator designers should consider this phenomenon, so that the area where the newborn lies in is the least reverberant.

Taking into account that the incubator is a closed box in which neonates spend a crucial part of their lives, ANSI/AAMI/IEC standards should also consider including requirements to limit the reverberation phenomenon within incubators.

## Data Availability Statement

The original contributions presented in the study are included in the article, further inquiries can be directed to the corresponding author.

## Author Contributions

VP-R conceptualized and designed the study. VP-R and DN-S carried out the acoustic measurements and supervised data collection. VP-R, DN-S, and FF-Z carried out the initial analyses and drafted the initial manuscript. VP-R, DN-S, FF-Z, RH-M, and EJ-M reviewed and revised the manuscript. All authors contributed to the article and approved the submitted version.

## Conflict of Interest

The authors declare that the research was conducted in the absence of any commercial or financial relationships that could be construed as a potential conflict of interest.

## Abbreviations

Sd: Standard deviation
NICU: Neonatal intensive care unit
*T*: Reverberation time
SPL: Sound pressure level
*T*_30_: Time in which the sound pressure level decays 30 dB multiplied by 2.
